# Longitudinal Effects of Cultural and Psychosocial Factors on Biomarkers of Cardiovascular Risk in Caregivers

**DOI:** 10.1093/geroni/igab046.008

**Published:** 2021-12-17

**Authors:** Samara Barrera Caballero, Rosa Romero Moreno Carlos Vara-García, María del Sequeros Pedroso Chaparro, Javier Olazarán, Brent Mausbach, Roland von Känel, Andrés Losada-Baltar

**Affiliations:** 1 Universidad Rey Juan Carlos de Madrid, Madrid, Madrid, Spain; 2 Universidad Rey Juan Carlos, Madrid, Madrid, Spain; 3 Universidad Autónoma de Madrid, Madrid, Madrid, Spain; 4 HGU Gregorio Marañón,, Madrid, Madrid, Spain; 5 University of California San Diego, La Jolla, California, United States; 6 University Hospital Zurich, Zurich, Zurich, Switzerland

## Abstract

Dysfunctional thoughts about caregiving (DTAC) and familism (i.e. familistic obligation) were associated with worse caregiver emotional and cardiovascular health in cross-sectional studies. The aim of this study was to longitudinally examine the effects of familism and DTAC on cardiovascular health, considering caregiver kinship adjusting for well-established predictors of cardiovascular health. Study participants were 80 family dementia caregivers. Individual interviews and collection of blood samples were conducted in three yearly assessments. Linear mixed (random effects) regression analysis was performed to examine longitudinal associations of familism, DTAC, and circulating levels of cytokine interleukin (IL)-6, a cytokine and biomarker of cardiovascular disease risk (CVD). Caregiver age, gender, alcohol consumption, body mass index (BMI), hours caring, frequency and reaction of behavioral problems and caregivers’ transitions were used as covariates. Results showed that increases in DTAC, in familism and higher caregiver age were independently and significantly associated with higher levels of IL-6 over time in the group of spousal caregivers. No significant effects were found for any of the other covariates in spousal caregivers. In contrast, increases in BMI and in frequency of behavioral problems were significantly associated with increases in IL-6 over time in adult child caregivers. No significant effects were found for any of the rest of predictors in adult child caregivers. Findings suggest that high level of obligation familism and DTAC may a profile of increased vulnerability for CVD in spousal caregivers. In contrast, problem behaviors of the care recipient may characterize adult child caregivers in terms of an increased CVD risk.

